# A Tyrosine-Rich Cell Surface Protein in the Diatom *Amphora coffeaeformis* Identified through Transcriptome Analysis and Genetic Transformation

**DOI:** 10.1371/journal.pone.0110369

**Published:** 2014-11-05

**Authors:** Matthias T. Buhmann, Nicole Poulsen, Jennifer Klemm, Matthew R. Kennedy, C. David Sherrill, Nils Kröger

**Affiliations:** 1 B CUBE Center for Molecular Bioengineering, Technische Universität Dresden, Dresden, Germany; 2 Department of Chemistry and Food Chemistry, Technische Universität Dresden, Dresden, Germany; 3 School of Chemistry and Biochemistry, Georgia Institute of Technology, Atlanta, Georgia, United States of America; Mount Allison University, Canada

## Abstract

Diatoms are single-celled eukaryotic microalgae that are ubiquitously found in almost all aquatic ecosystems, and are characterized by their intricately structured SiO_2_ (silica)-based cell walls. Diatoms with a benthic life style are capable of attaching to any natural or man-made submerged surface, thus contributing substantially to both microbial biofilm communities and economic losses through biofouling. Surface attachment of diatoms is mediated by a carbohydrate- and protein- based glue, yet no protein involved in diatom underwater adhesion has been identified so far. In the present work, we have generated a normalized transcriptome database from the model adhesion diatom *Amphora coffeaeformis*. Using an unconventional bioinformatics analysis we have identified five proteins that exhibit unique amino acid sequences resembling the amino acid composition of the tyrosine-rich adhesion proteins from mussel footpads. Establishing the first method for the molecular genetic transformation of *A. coffeaeformis* has enabled investigations into the function of one of these proteins, AC3362, through expression as YFP fusion protein. Biochemical analysis and imaging by fluorescence microscopy revealed that AC3362 is not involved in adhesion, but rather plays a role in biosynthesis and/or structural stability of the cell wall. The methods established in the present study have paved the way for further molecular studies on the mechanisms of underwater adhesion and biological silica formation in the diatom *A. coffeaeformis*.

## Introduction

Diatoms are a large group of single-celled microalgae that are ubiquitously present in water habitats, and are among the most prolific biological primary producers in the oceans [1]. The hallmark of diatoms is that each cell is encased by a wall made of intricately patterned SiO_2_ (silica). Diatoms are widely studied due to their importance for ocean ecosystems, their physiological capabilities, their complex evolutionary history, and their ability to adhere to any natural or man-made surface underwater. The colonization of submerged surfaces (“biofouling”) by bacteria, microalgae (including diatoms), and multicellular organisms (e.g., barnacles, mussels and macroalgae) can lead to the development of biofilms that are several centimeters thick [2]. Biofouling causes enormous costs world-wide due to the increase in hydrodynamic drag of ships, and damage to aquaculture equipment [3], [4]. Therefore, substantial efforts are being spent on developing environmentally friendly agents that prevent biofilm formation by inhibiting the initial attachment of bacteria and diatoms [5], [6]. On the other hand, the adhesive components produced by diatoms provide a paradigm for the development of underwater glues for numerous applications in technology and medicine [7]. For both, the prevention of diatom adhesion and the development of underwater glues, it is necessary to identify the adhesive biomolecules of diatoms and understand their molecular mechanism of adhesion.

While little is known about the attachment of microalgae, several extracellular proteins required for the adhesion of bacterial biofilms have been identified. These include amyloid-fibers that provide biofilm matrix cohesiveness [8], and flagella that are required for bacterial attachment to abiotic surfaces [9]. Diatoms, however, adhere with fundamentally different mechanisms that do not involve flagella but rather adhesive strands, which are secreted through a slit-like cell wall opening termed “the raphe” [10]. Recently, it has been demonstrated that diatom adhesive material is composed of both protein and carbohydrate, and their amino acid and monosaccharide compositions have been determined [11]. However, so far no sequence information from these macromolecules has been obtained.

To date, the molecular mechanisms of underwater adhesion have been studied in most detail in animals, particularly the marine mussel *Mytulis edulis*. Mussels attach to surfaces via macroscopic fibers that contain tyrosine-rich proteins at their sticky end (i. e., the mussel foot). In many mussel foot proteins tyrosine residues have been post-translationally hydroxylated to 3,4-dihydroxyphenyl-L-alanine (Dopa) [12]. The presence of Dopa seems to play an important role in both structural integrity of the filaments and underwater adhesion to surfaces by forming covalent cross-links and coordination bonds with metal ions, as well as by forming hydrogen bonds with the surface. Also the adhesive proteins of other organisms, like polycheates, invertebrates, turbellarians, hydroids and tunicats contain significant amounts of Dopa (for a review, see [13]).

The pivotal role of Dopa-rich proteins in underwater surface adhesion of lower animals has prompted the question as to whether diatoms employ similar proteins. This question has been addressed in the present work using a bioinformatics-based approach. *Amphora coffeaeformis* was chosen for these studies, because it is one of the most common biofouling diatoms, and it has been used in many studies as a model organism for underwater bioadhesion [14]–[16]. Previously, *A. coffeaeformis* had not been investigated on a molecular level, and thus neither genome nor transcriptome data were available for this species at the onset of this study. Through the present work we have made *A. coffeaeformis* amenable for investigations on the molecular mechanism of underwater adhesion through establishing a transcriptome database and a method for its molecular genetic transformation. These tools have then been employed to identify *A. coffeaeformis* proteins with similarities to mussel adhesion proteins, and first steps have been taken towards their functional characterization.

## Materials and Methods

### Strains and culture conditions

Cultures of *Amphora coffeaeformis* (C. Agardh) Kuetzing clone CCMP126 were grown on the bottom of 1 Liter Fernbach flasks in an artificial seawater medium (coined NEPC medium) according to the North East Pacific Culture Collection (http://www3.botany.ubc.ca/cccm/NEPCC/esaw.html). Cultivation conditions were 18°C and constant light at an intensity of 40–60 µmol photons m^−2^ s^−1^, using cool white and warm white fluorescent tubes as light source. Bacteria-free (axenic) cultures were obtained by a 3-day treatment with antibiotics (1 mg mL^−1^ penicillin, 0.5 mg mL^−1^ streptomycin) and subsequent recovery under antibiotic-free conditions.

### RNA isolation and transcriptome sequencing

Total RNA was extracted from cells that were attached to and actively moving on polystyrene petri dish surfaces using the RNAqueous Micro kit (Ambion, Carlsbad, CA, USA) by applying the lysis buffer directly to the petri dish surface after decanting the culture medium. The resulting total RNA (100 µg) was sent to Eurofins Genomics (Hunstville, AL, USA) for generation of a normalized random primed cDNA library and subsequent sequencing. Briefly, first strand cDNA was synthesized from isolated polyA^+^ mRNA using random hexamers with the subsequent ligation of 3′ adapters. Second strand synthesis was performed using the 3′ adapter sequence. The cDNA library was then size-fractionated and normalized. Subsequently, the normalized cDNA was sequenced by Roche GS FLX technology using Titanium series chemistry (half a plate). The contig assemblies were performed by Eurofins Genomics. The transcriptome sequence data have been deposited in the NCBI Sequence Read Archive (SRA) under accession number SRP046053.

### Bioinformatics analysis

The database screening tool was written in Python, utilizing the common gateway interface (CGI) to interact with the web page at the URL http://vergil.chemistry.gatech.edu/cgi-bin/proteomics.py.

The *A. coffeaeformis* transcriptome sequence database was translated in all three forward frames and compiled in FASTA format. Once the composition and domain size are selected, the database is read in and each sequence is analyzed for composition and checked against all the composition requirements. If a domain length requirement of *n* is given, the first *n* amino acids are checked against the composition requirements. If the requirements are not met, then the first amino acid in the window being analyzed is subtracted out, the next amino acid is added in, and the requirements are checked again.

### Construction of expression vector pPhaT1/YFP+fcpA/nat

A *Phaeodactylum tricornutum* expression vector was generated to allow for the single step construction of genes that encode fusion proteins carrying C-terminal YFP. In the first step the *eyfp* gene was amplified using the sense primer 5′-**GAA TTC** TAC GTA *GCA TGC* ***TCT AGA*** GGC GGA ATG GTG AGC AAG GGC GAG G-3′ and the antisense primer 5′-**AAG** **CTT** TTA CTT GTA CAG CTC GTC CAT-3′, which introduced a single copy of each of the following restriction sites *EcoR*I (bold), *SnaB*I (underlined), *Sph*I (italics), *Xba*I (bold, italics), and *Hind*III (bold, underlined). The resulting PCR products were introduced into pJet1.2 (Thermo Fisher Scientific; Waltham, MA, USA), sequenced, and then subcloned into the *EcoR*I and *Hind*III sites of pPhaT1/nat, which was kindly provided by Kirk Apt [17]. This generated the plasmid pPhaT1/YFP, which also contains the *ble* gene for resistance to zeocin under the control of the fcpB promoter (fcpB/*ble*). The fcpB/*ble* gene fragment was excised from pPhaT1/YFP using *Pst*I and *Xho*I (blunted using T4 DNA polymerase), and replaced by the fcpA/*nat* gene fragment derived from pPhaT1/*nat* by digestion with *Xho*I (blunted with T4 DNA polymerase) to generate pPhat1/YFP+fcpA/*nat*. This vector contains a short multiple cloning site (*SnaB*I, *Sph*I, *Xba*I) that allows for the generation of C-terminally tagged YFP fusion proteins.

### Construction of fusion genes

Total RNA isolation, synthesis of a cDNA library attached to oligo(dT)_25_ magnetic beads (Invitrogen, Carlsbad, CA, USA), and rapid amplification of cDNA ends (RACE PCR) was performed as described previously [18]. The resulting PCR products were cloned into pJet1.2 and sequenced by Eurofins Genomics (Ebersberg, Germany). Genomic DNA was isolated according to an established protocol [19]. For the generation of C-terminal YFP fusions genes the full length gene was amplified from genomic DNA using oligonucleotide primers that introduced either a *SnaB*I or *EcoR*V restriction site at the 5′-end and an *Xba*I restriction site at the 3′-end of the gene. The genes were amplified using Phusion DNA polymerase (Thermo Fisher Scientific), and the resulting PCR products were cloned into pJet1.2 and sequenced. The pJet1.2/AC genes were subsequently digested with the appropriate restriction enzyme and cloned into the SnaBI/XbaI site of pPhat1/YFP+fcpA/*nat*. Due to the apparent instability of the *AC4076-YFP* fusion gene it was necessary to use SURE2 *E. coli* cells (Agilent; Waldbronn, Germany) for its cloning. All other cloning procedures were performed using DH5α.

### Transformation of Amphora coffeaeformis

Exponentially growing cells were harvested and concentrated by centrifugation for 5 min at 3,220 g. A total of 10^8^ cells were plated in a 5 cm circle in the center of a NEPC medium agar plate (1.5% agar; Difco, Becton, Dickinson and Company, UK), and allowed to dry. Bombardment with DNA-coated tungsten particles was performed using the Biolistic PDS-1000/He particle delivery system (Bio-Rad, Hercules, CA, USA) as previously described [20]. DNA-coated tungsten particles were prepared by mixing 300 µg of tungsten particles of different diameter (i. e., 40 nm from Chempur, Karlsruhe and 400 nm, 700 nm, 1100 nm from Bio-Rad, München, Germany) with 5 µg of circular plasmid DNA using the CaCl_2_-spermidine method [21]. For bombardment of the cells, agar plates were placed at a distance of 7 cm using either 1,500 psi rupture disks (1,400–1,600 psi) or 2,000 psi rupture disks (1,800–2,100 psi) (Bio-Rad, München, Germany). Immediately after bombardment the agar dishes were covered with liquid NEPC medium and incubated for 16 h under constant illumination. Subsequently, cells were spread on selective plates (5×10^6^ cells per plate) containing 300 µg mL^−1^ of nourseothricin (clonNat; Werner Bioagents, Jena, Germany), and incubated at cultivation conditions either in continuous light or at a 14 hours light:10 hours dark rhythm.

### Fluorescence and bright field microscopy

To screen for fluorescent transformant cells, clones from selective agar plates were inoculated into NEPC medium containing 300 µg mL^−1^ of nourseothricin in a 96-well glass bottom optical plate (Corning, Kaiserslautern, Germany) and incubated for 24–48 h prior to observation with an Axioplan 200 epifluorescence microscope (Zeiss, Jena, Germany) equipped with a YFP band pass filter set (EX: HQ500/20x; BS: Q515lp; EM: HQ535/30m; Chroma, Bellows Falls, VT, USA). Confocal fluorescence microscopy images were acquired with an inverted laser scanning microscope LSM 780/FLIM equipped with 32-channel GaAsP spectral detector and a multiline argon laser (458, 488, 514 nm) (Zeiss, Jena, Germany) using an alpha Plan-Apochromat 63x/1.46 Oil Korr M27 objective (Zeiss) and the Zen software (2011 version; Zeiss). YFP fluorescence was excited with the argon laser at 488 nm and detected using a 535/30 nm bandpass filter. During epifluorescence microscopy isolated cell walls were imaged using an Axioplan 200 epifluorescence microscope equipped with an eYFP 535/30 nm bandpass filter set (Zeiss).

Immunofluorescence images were acquired using an NSTORM laser microscope equipped with an Andor Ixon Ultra 897 camera (Nikon Instruments Europe B.V., Amsterdam, Netherlands) and a CFI TIRF Apochromat 100x/1.49 Oil objective (Nikon Instruments Europe B.V.). YFP fluorescence was excited at 488 nm and detected using a bandpass filter (502–548 nm, F37–521; AHF Analysentechnik, Tübingen, Germany), Alexa Fluor 647 fluorescence was excited at 647 nm and detected using a bandpass filter (663–738 nm, F47–700, AHF Analysentechnik).

Diatom trails were stained with 1 mg mL^−1^ 1-Ethyl-2-[3-(1-ethylnaphtho[1,2-d]thiazolin-2-ylidene)-2-methylpropenyl]naphtho[1,2-d]thiazolium bromide, 3,3′-Diethyl-9-methyl-4,5,4′,5′-dibenzothiacarbocyanine bromide (Stains-All; Sigma-Aldrich) in formamide for 15 min, washed excessively with H_2_O, and then analyzed with an Axioplan 200 epifluorescence microscope (Zeiss) in the bright field mode as previously described [11].

### Scanning electron microscopy

To prepare *A. coffeaeformis* biosilica for electron microscopy, cells were extracted twice in methanol (95°C) for 15 minutes each, and then washed three times with H_2_O. The methanol-extracted cell walls were then treated with 1% SDS at 95°C for 30 min, washed four times with H_2_O, and dried onto 0.5′′ aluminium stubs (Agar Scientific, Stansted, UK). Electron microscopy was performed with a JSM7500F SEM (Jeol, München, Germany) at 10 kV acceleration voltage.

### Cell wall isolation

A total of 3×10^9^ *A. coffeaeformis* cells were harvested by decanting ∼90% of the culture supernatant, resuspending the attached biofilm in the remaining supernatant using a cell scraper, and pelleting the cells by centrifugation for 5 min at 3220 g. The pelleted cells were thoroughly resuspended in buffer A (100 mM Tris-acetate pH 8, 50 mM EDTA, EDTA-free Pierce protease inhibitor (Thermo Fisher Scientific)), followed by extraction with 1 v/v-% Triton X-100 in buffer A at room temperature for 10 min. The cells were pelleted by centrifugation for 5 min at 3220 g and washed by resuspending the pellet in buffer B (100 mM Tris-acetate pH 8). This washing procedure was repeated three times. Finally, the cells were extracted with 1 w/v-% SDS in buffer B at room temperature for 10 min, centrifuged and washed in buffer B as described above, and extracted with 50% acetone until the material was colorless. All extractions were supported by vortexing and gentle sonication in a water bath at room temperature. Finally the cell walls were washed three times with buffer B as above.

### Preparation of cell wall extracts

A total of 1.5×10^9^ cell walls were resuspended in 2 mL SDS-extraction buffer (1 w/v-% SDS in buffer B) and shaken for 10 min at 55°C and 1,400 rpm in an thermomixer (Eppendorf AG, Hamburg, Germany). After pelleting by centrifugation at room temperature for 10 min at 4,000 g, the SDS extract was collected, the pellet resuspended in the same extraction buffer, and incubated under the same conditions. This procedure was performed three times in total, and the extracts were combined. The final pellet was washed twice by resuspension in buffer B and then centrifuged as above. The supernatants of the washing steps were combined with the supernatants of the extraction steps yielding the SDS cell wall extract. The SDS cell wall extract was diluted with H_2_O to a final concentration of 0.1% SDS, and then concentrated by ultrafiltration (10 kDa MWCO; Amicon, Millipore, Darmstadt, Germany) to approximately one eightieth of the original volume. The original volume was restored by adding 200 mM ammonium acetate, and subsequently concentrated again to approximately one eightieth of the volume. The dilution-concentration cycles were repeated four times (i. e., until the SDS was largely removed) to a final volume of 300 µL.

For extraction with ammonium fluoride, 1.5×10^9^ cell walls were resuspended in 6 mL 10 M NH_4_F, adjusted to pH 4–5 by drop-wise addition of 6 M HCl, and incubated for 30 min at room temperature. After centrifugation for 15 min at 3,220 g at 4°C, the extraction procedure was repeated. The ammonium fluoride insoluble material was finally pelleted as before, washed with 200 mM ammonium acetate and pelleted again. All supernatants were unified, desalted and concentrated by ultrafiltration (10 kDa MWCO; Amicon, Millipore) as described above.

### Western Blot

Protein extracts from 2.3×10^8^ cell walls were separated on 10% Laemmli gels by gel electrophoresis, and transferred to nitrocellulose membranes (Protran BA 85; Whatman, GE Healthcare Europe, Freiburg, Germany) using a wet-blotting system (Bio-Rad, München, Germany) at 100 V for one hour. Towbin buffer [22] (25 mM Tris, 192 mM glycine) with 20 v/v-% methanol and 0.05% SDS was used for the transfer. Western Blots were probed with polyclonal Rabbit anti-GFP antibody (diluted 1∶2,000 in Roti Block; referred to as “anti-YFP antibody”, Cat. No 632592, Living Colours full-length A.v. polyclonal; Clontech, Mountain View, CA, USA) and secondary antibody peroxidase conjugate (diluted 1∶10,000 in Roti Block; Anti-Rabbit IgG, A0545; Sigma-Aldrich, München, Germany). After 5 min development with chemiluminescent substrate (SuperSignal West Pico; Thermo Fisher Scientific) signals were detected on X-ray film (CL- XPosue Film; Thermo Fisher Scientific).

### Immunolabeling

For immunolabeling of cell walls and ammonium fluoride insoluble material, the samples were resuspended in 1× blocking agent (Roti Immunoblock, Carl Roth, Karlsruhe, Germany) and incubated for 1 hour at room temperature with constant gentle shaking. Subsequently, anti-YFP antibody (see “Western Blot”) was added to a final dilution of 1∶300, and the samples were incubated with gentle shaking for an additional 1 hour. Afterwards the samples were pelleted by centrifugation (10 min at 3,220 g) and resuspended in 1× blocking agent. The centrifugation-resuspension procedure was repeated two more times, and after the last step Alexa Fluor 647-labeled anti-rabbit IgG (F(ab′)_2_ (H+L) fragment developed in goat; Invitrogen, Germany) was added to a final dilution of 1∶8,000 in 1× blocking agent. Before imaging, the samples were washed at room temperature by centrifugation and resuspenion three times in 1x blocking agent, and three times in 100 mM Tris-acetate pH 8.

## Results

### Generation of a transcriptome database for Amphora coffeaeformis

RNA was isolated from *A. coffeaeformis* cells that were adhered to polystyrene petri dishes, and a normalized *A. coffeaeformis* cDNA library was generated. Sequencing of the cDNA library yielded a total of 659,065 raw reads, with an average length of 341 bp. After trimming for the adaptors and primer sequences, 29,306 sequences were removed due to their short length resulting in 568,626 high quality (HQ) reads. The HQ reads were assembled into 41,824 contiguous sequences (contigs) ranging in size from 41 to 8,684 bp, with an average length of 799±509 bp.

### Bioinformatics search for candidate adhesion proteins in A. coffeaeformis

Screening of the *A. coffeaeformis* transcriptome database for diatom proteins with sequence similarity to underwater adhesion proteins from mussels and the sandcastle worm was unsuccessful. Therefore, in an extension of our previous work [23], we have developed here a bioinformatics analysis tool that enables the in-depth screening of sequence databases for proteins based on amino acid composition rather than amino acid sequence. The tool allows for the identification of proteins that exhibit amino acid compositions of interest within a defined sequence domain. Both amino acid composition (in mol-%) and domain size (in number of amino acids) are freely selectable. Additionally, proteins can be screened simultaneously for the presence of an N-terminal signal peptide (required for most secreted proteins) according to the SignalP algorithm [24]. This new amino acid composition-based database screening tool is available at a publicly accessible website (http://vergil.chemistry.gatech.edu/cgi-bin/proteomics.py).

By applying this bioinformatics tool, the transcriptome of *A. coffeaeformis* was screened for putative adhesion proteins based on amino acid composition similarity to mussel foot proteins FP-3 and FP-5. Both proteins are present in the adhesive pads, and are believed to be directly involved in surface adhesion [25], [26]. The unmodified FP-3 and FP-5 polypeptides exhibit molecular masses of 7.5–8.9 kDa and 8.9–12.2 kDa respectively, and a high content of tyrosine (Y>10 mol-%) [27]. Protein FP-1 is a coating rather than adhesive protein, yet it has also a high tyrosine content (up to 15 mol-%) and exhibits adhesive properties when the tyrosine residues are converted to Dopa [26], [27]. Additional FP-1 characteristics are a high content of lysine (K>11 mol-%), and a highly repetitive sequence structure. An essential selection criterion in screening the diatom transcriptome database for candidate adhesion proteins was the presence of an N-terminal signal peptide, because the diatom adhesion proteins are expected to be assembled in and transported through the secretory pathway [28]. When screening the *A. coffeaeformis* transcriptome database for proteins with high tyrosine and high lysine content, five predicted proteins were retrieved that matched the search criteria (Table 1). The cDNA sequences of the encoding genes were validated by reverse transcriptase (RT) PCR (including 5′- and 3′-RACE PCR), except for Ac203 for which 3′-RACE PCR failed. The polypeptide sequences of the five proteins and the sequences of oligonucleotide primers used for RT PCR are shown in Table S1 and Table S2, respectively.

**Table 1:** Candidate adhesion proteins identified by an amino acid composition-based bioinformatics screen of the *A. coffeaeformis* transcriptome database. n. a.  =  not applicable.

Search criteria	Domain size (amino acids)	Protein ID
>10 mol-% Y	n. a.	AC1077, AC4076, AC714
>8 mol-% Y and>20 mol-% K	50–200	AC203
>10 mol-% Y and>20 mol-% K	100–300	AC3362

Interestingly, the putative adhesion proteins from *A. coffeaeformis* share additional features with mussel adhesion proteins that had not been selected for in the bioinformatics screen. These features are a high glycine content (9.2 mol-% for AC203; 23.4 mol-% for AC1077; 16.3 mol-% for AC714), and a high proline content (23.4 mol-% for AC1077; 14.3 mol-% for AC3362) (Table S3). Standard BLAST searches in the NCBI database using the complete sequences of the Y-rich proteins from *A. coffeaeformis* did not reveal homologous proteins in diatoms or other organisms. However, the Position-Specific Iterated (PSI) BLAST algorithm revealed that certain segments of proteins AC4076, AC714, and AC3362 exhibited sequence similarity to proteins from other organisms. A tyrosine- and histidine-rich stretch of AC4076 was similar to a putative adhesion protein from the fungus *Naumovozyma dairenensis* (29% identity over 103 amino acids, E-value: 3e-43; Table S4, Figure S1). A tyrosine-, lysine- and glycine-rich region of AC714 showed high similarity to a domain from a putative collagen alpha-1V chain from the insect *Danaus plexippus* (41% identity over 179 amino acids, E-value: 3e-36, Table S4, Figure S1). A repetitive proline- and lysine-rich region of AC3362 that also exhibited a high tyrosine and threonine content was highly similar to a domain from a fungal protein of unknown function (67% identity over 185 amino acids, E-value: 1e-68; Table S4, Figure S1). However, PSI-BLAST analysis did not detect sequence similarity of regions from the Y-rich proteins to any domains from the mussel foot proteins.

**Figure 1:**
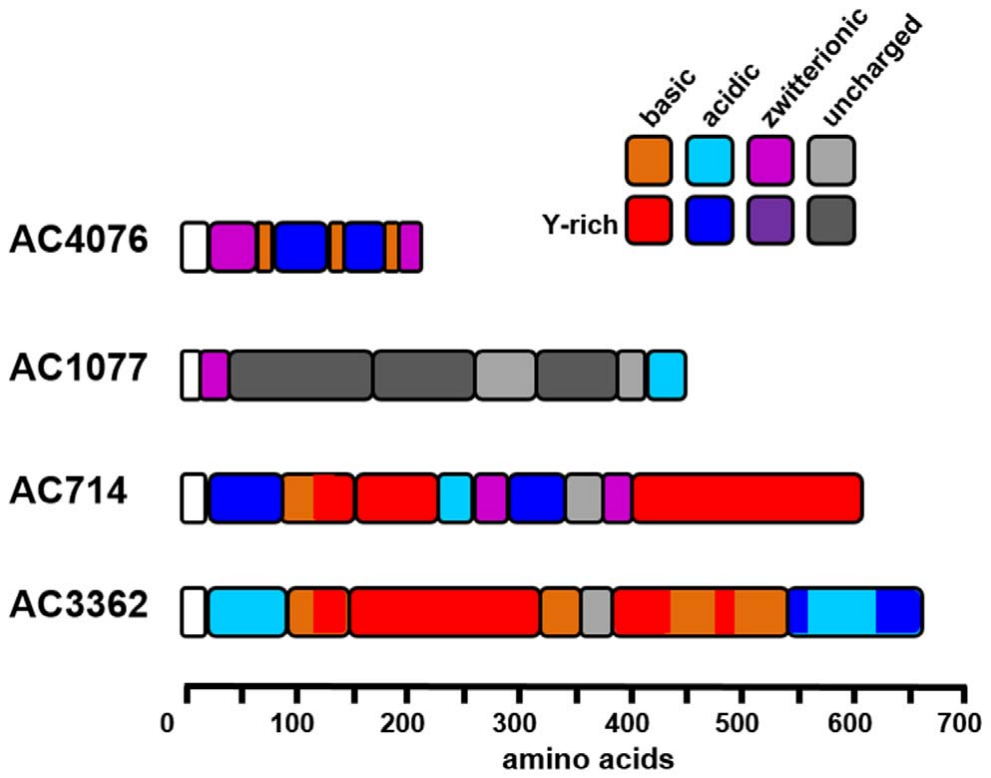
Schematic primary structures of the Y-rich proteins from *A. coffeaeformis*. Domains with a high abundance of charged amino acids (≥25 mol-%) are colored. Red color indicates modules with a surplus of positively charged amino acids (mol-% ratio D+E: K+R≥2), blue modules are dominated by negatively charged amino acids (mol-% ratio K+R: D+E ≥2), and purple color indicates zwitterionic modules in which negatively and positively charged amino acids are almost balanced (mol-% ratio K+R: D+E>0.8 or <1.2). Gray modules are essentially devoid of charged amino acids (<10 mol-%). In all modules dark color indicates regions with a tyrosine content ≥6 mol-%. Note that the average contents of tyrosine in all proteins in the Swiss-Prot database is 2.9%, of D+E is 12.2%, and of K+R is 11.4% [46]. White modules indicate the signal peptide.

As we were unable to obtain information on the full length cDNA sequence of AC203 further analysis of this protein was discontinued. The amino acid sequences of the remaining four Y-rich proteins (AC1077, AC4076, AC714, AC3362) exhibit a modular structure which often contain repetitive peptide motifs (Figure 1, File S1). In most domains except for those of AC1077 charged and/or polar amino acids dominate (>25 mol-%), giving AC4076, AC714, and AC3362 a strongly hydrophilic character despite the high tyrosine content. In all four proteins, lysine rather than arginine residues account for almost all positively charged amino acid residues. There are also several proline-rich modules, in which proline residues account for 16–54 mol-% of the amino acid residues.

To gain the first insight into the function of the *A. coffeaeformis* Y-rich proteins, we intended to overexpress them as fluorescent fusion proteins in *A. coffeaeformis*, and study their locations *in vivo*. To enable such experiments, it was first necessary to establish a genetic transformation method for this diatom species.

### Genetic transformation of Amphora coffeaeformis

To date, all routinely used methods for the genetic transformation of diatoms rely on microparticle bombardment (termed biolistic transformation). In this method tungsten particles are coated with plasmid DNA containing both the gene of interest and an antibiotic resistance gene. In most cases endogenous diatom specific promoters are used for driving the expression of both genes, but also heterologous diatom promoters or even non-diatom promoters have been shown to work in some cases [29]–[32]. Promoters from *A. coffeaeformis* genes are not available due to the complete lack of sequence information from the genome of this species. Therefore, transformation experiments had to be been performed using promoters from other diatom species (see below).

Growth of *A. coffeaeformis* wild type cells in liquid culture and on agar plates can be completely inhibited by the antibiotics zeocin (at ≥600 µg mL^−1^) or nourseothricin (at ≥300 µg mL^−1^). Therefore, for biolistic transformation experiments with *A. coffeaeformis* the *ble* gene (resistance to zeocin) and the *nat* gene (resistance to nourseothricin) were chosen as selection markers. Diatom specific expression vectors containing the antibiotic resistance genes under control of the *fcp* promoters from the diatoms *Phaeodactylum tricornutum*, *Cylindrotheca fusiformis*, and *Thalassiosira pseudonana* (pPhat1/*nat*, pCfcp/*ble*, Tpfcp/*nat*, Tpfcp/*ble*) [17], [20], [33] were coated onto tungsten microparticles, and used in biolistic transformation experiments. The promoters from all three diatom species appeared to be functional in *A. coffeaeformis* as indicated by the growth of *A. coffeaeformis* transformant clones on both on agar plates, and in liquid medium in the presence of antibiotic concentrations that were lethal to the wild type cells.

**Figure 2:**
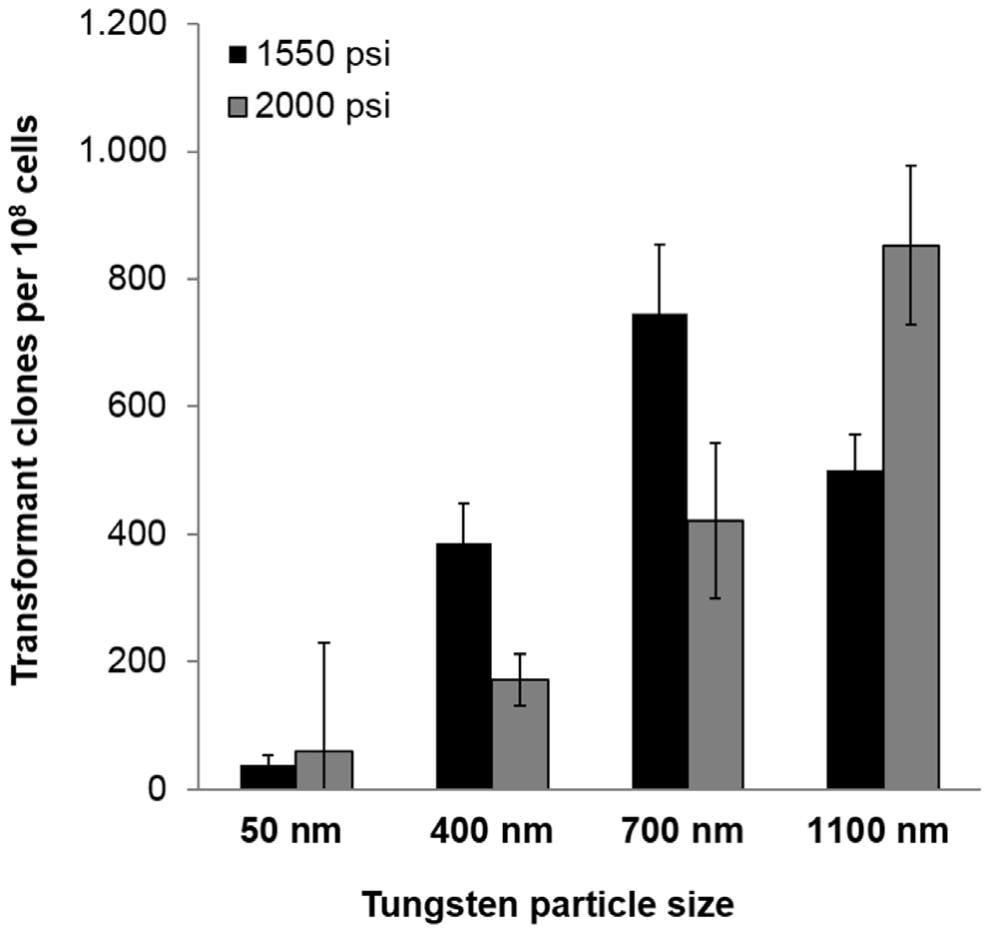
Influence of tungsten particle size and acceleration pressure on transformation efficacy. Tungsten particles of the indicated sizes were coated with plasmid DNA and shot at plated *A. coffeaeformis* cells using a helium pulse of 1550 psi (black bars) or 2000 psi (grey bars). Transformed clones were quantified by counting the number of colonies that grew on agar plates containing the antibiotic nourseothricin.

The highest number of antibiotic resistant *A. coffeaeformis* clones was obtained with the *P. tricornutum* fcp promoter (pPhat1) in combination with the *nat* resistance gene. Therefore, pPhat1/*nat* was used as the selection marker in all subsequent transformation experiments. However, even with pPhat1/*nat* the efficacy of genetic transformation was rather low, yielding a maximum of only 50 antibiotic resistant cells per 10^8^ bombarded cells. Through systematic variation of experimental conditions (i. e., tungsten particle size, particle acceleration pressure, and cultivation conditions) the transformation efficacy could be drastically improved consistently yielding 800 antibiotic resistant cells per 10^8^ bombarded cells (Figure 2). This yield is even higher than in currently used transformation protocols for *T. pseudonana* or *P. tricornutum*, which generate up to 400 transformant clones per 10^8^ cells [17], [20].

After spreading the cells on antibiotic containing agar plates, cultivation in a 10∶14 hour light:dark cycle rather than continuous light appeared to be beneficial (data not shown). Tungsten particles of average diameters of 700 nm and 1,100 nm were more effective than smaller particles (Figure 2), while lower particle acceleration (pressure of 1,550 psi) proved to be more effective except for the largest tungsten particles (Figure 2).

### Localization of AC3362

**Figure 3:**
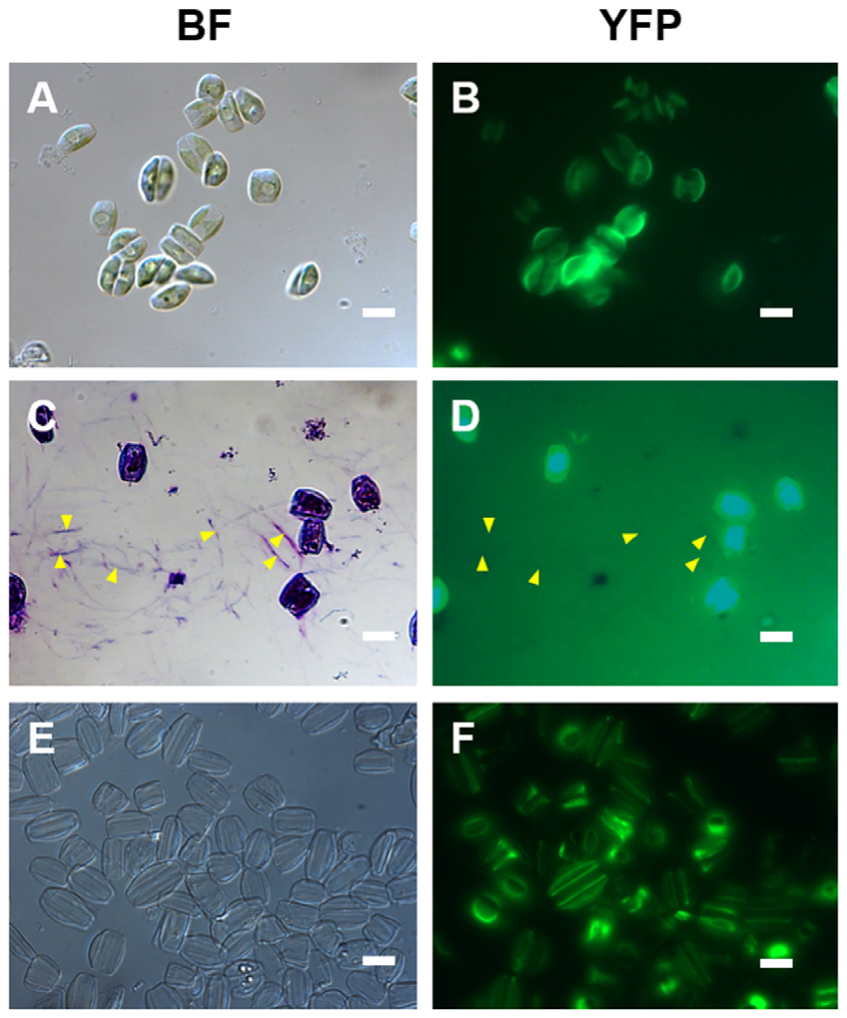
Localization of AC3362-YFP in *A. coffeaeformis*. (**A, C, E**) Bright field microscopy images, and (**B, D, F**) corresponding epifluorescence microscopy images. (**A, B**) Live cells. (**C, D**) Cells after treatment with the polycationic dye ‘Stains-All’. For orientation some trails are labeled by arrowheads. Image (**D**) was deliberately overexposed to check for YFP fluorescence in the trails. (**E, F**) Isolated cell walls. Bars = 10 µm.

For four of the five candidate adhesion proteins full-length cDNA sequences could be obtained by RACE PCR (i. e., AC1077, AC714, AC3362, AC4076). We constructed expression plasmids in which each of the four *A. coffeaeformis* genes was fused to the YFP gene under the control of the *P. tricornutum* fcpA promoter (pPhat1), which were then introduced into *A. coffeaeformis* using the transformation method described above. At least 27 nourseothricin-resistant transformant clones were analyzed by fluorescence microscopy for each expression construct (Table S5). Only transformants harboring the AC3362-YFP fusion gene exhibited YFP fluorescence in the cells (Figure 3A, B), transformation with the other YFP fusion genes (i. e., AC1077, AC714, AC4076) did not result in fluorescent clones (data not shown). None of the AC3362-YFP transformant cells exhibited YFP fluorescence in the adhesive material that is deposited on the glass slides as characteristic trails (Figure 3C, D). The AC3362-YFP fusion protein appeared to be located at the periphery of the cell (Figure 3A, B) suggesting that it is a cell wall associated protein. This was confirmed by isolating the cell walls, which still exhibited strong YFP fluorescence (Figure 3E, F).

**Figure 4:**
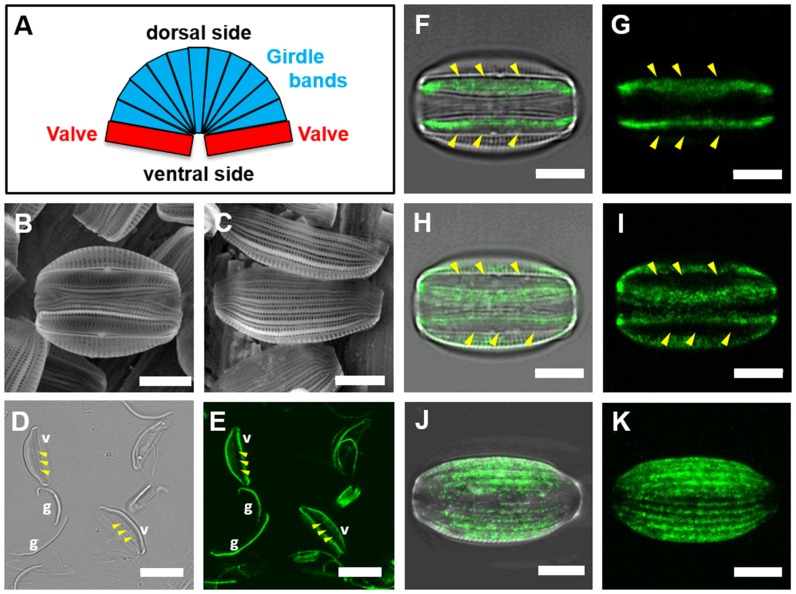
Localization of AC3362-YFP within the cell wall. (**A**) Schematic of an *A. coffeaeformis* cell wall. (**B, C**) Scanning electron micrographs of *A. coffeaeformis* cell walls in (**B**) ventral view, and (**C**) dorsal view. (**D, E**) Sonicated cell walls imaged with bright field microscopy (**D**), and confocal fluorescence microscopy (**E**). Valves (v) and girdle bands (g) are labeled. (**F-K**) Confocal fluorescence microscopy images of isolated cell walls in (**F-I**) ventral view, and in (**J, K**) dorsal view. The images represent 2D projections of confocal Z-stacks of the cell wall in contact with the surface-substratum (**F, G**), around the raphe region (**H, I**), and throughout the girdle band region (**J, K**). (**F, H, J**) show overlays of confocal fluorescence microscopy images and the corresponding bright field images, while (**G, I, K**) show the confocal fluorescence microscopy images only. In (**D-I**) the positions of the raphes are indicated by a triplet of arrowheads. Bars: (B, C, F-K) = 5 µm, (D, E) = 10 µm.

To investigate the localization of AC3362-YFP in the cell wall in more detail, epifluorescence and confocal fluorescence microscopy analysis of both intact and fragmented cell walls were performed. Like with all other diatoms *A. coffeaeformis* cell walls are composed of two silica plates, each termed valve, which are connected by circular silica strips, termed girdle bands. In most diatoms the two valves are located on the opposing poles of the cell, but the genus *Amphora* is characterized by the two valves being positioned on the same side of the cell (i. e., the ventral side; Figure 4A, B). As a consequence the girdle bands of *A. coffeaeformis* are wedge shaped, being narrower on the ventral side than on the opposing side (i. e., the dorsal side; Figure 4A-C). When isolated cell walls were treated with controlled doses of ultrasound, the girdle bands became separated from the valves (Figure 4D). Epifluorescence microscopy revealed that the AC3362-YFP fusion protein was present both in the girdle bands and the valves (Figure 4D, E). In the valves the YFP fluorescence appeared to be most abundant at the edges and significantly weaker in the central region around the so-called raphe (Figure 4D, E). The raphe contains a longitudinal slit through which the adhesive material is secreted [34]. Confocal fluorescence microscopy confirmed that the AC3362-YFP fusion protein was present almost everywhere on the valve surface except for the region of the raphe (Figure 4F-I). The absence of AC3362-YFP in the raphe, through which the adhesive material is being secreted, is consistent with the absence of this fusion protein in the trails that contain the adhesive material (see Figure 3C, D). The dorsal side of intact isolated cell walls contains only girdle bands and exhibits a striated fluorescent pattern that is congruent with the longitudinal axes of the girdle bands (Figure 4J, K).

### Characterizing the Biosilica Association of AC3362-YFP

**Figure 5:**
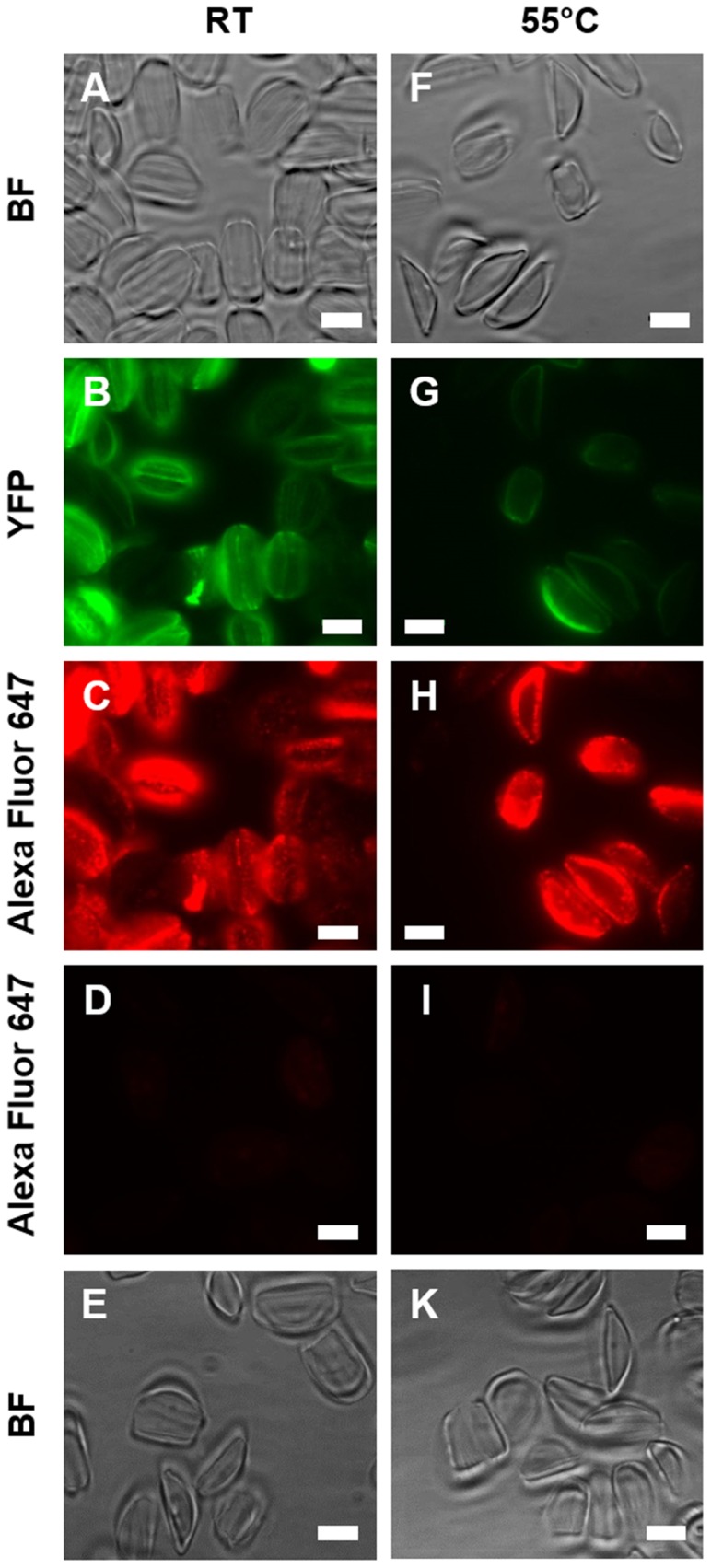
Biosilica association of AC3362-YFP. Isolated cell walls (RT) and isolated cell walls after hot SDS teatment (55°C) were analyzed by bright field microscopy (BF), by direct fluorescence microscopy (YFP), and by indirect immunofluorescence microscopy (Alexa Fluor 674) using an anti-YFP primary antibody and an Alexa Fluor 647-labeled secondary antibody. (**A-C**) Cell walls from a transformant clone expressing AC3362-YFP, and (**F-H**) cell walls from the same clone after extraction with a hot SDS solution. Cell walls from wild type (**D, E**) before and (**I, K**) after extraction with a hot SDS solution. Bars = 5 µm.

The results presented above strongly suggest that AC3362 is a component of the *A. coffeaeformis* cell wall rather than a protein involved in surface adhesion. The amino acid sequence of AC3362 does not exhibit any similarities to previously characterized diatom cell wall proteins, which are frustulins, pleuralins, silaffins, silacidins, cingulins, and p150 [23], [35]–[37]. However, regarding the high content of lysine (15.5 mol-%) and tyrosine (8.0 mol-%) residues, AC3362 resembles the cingulins of the diatom *T. pseudonana* [23] and the silaffin-1 peptides from *C. fusiformis* [38]. Cingulins and silaffins are tightly associated with the biosilica, and thus cannot be extracted from the cell walls even when using solutions of SDS at elevated temperature [23], [35], [39]. To determine the stability of the interaction between AC3362-YFP and the biosilica, isolated cell walls were treated with 1 w/w-% SDS at 55°C and YFP-fluorescence intensity of the cell walls was monitored by epifluorescence microscopy. After this treatment the YFP fluorescence in the cell walls was substantially decreased seemingly indicating that the majority of AC3363-YFP became extracted (compare Figures 5B and G). However, isolated cell walls before and after treatment with hot SDS solution exhibited comparable fluorescence intensity following indirect immunofluorescence labeling using primary antibodies against YFP and an Alexa Fluor 647-labeled fluorescent secondary antibody (compare Figures 5C and H). Wild type cells walls exhibited no fluorescence following immunolabeling (Figure 5D, E, I, K) demonstrating the specificity of the immunolabeling procedure. Altogether, the immunolabeling data indicate that most, if not all, of the AC3362-YFP fusion protein is still present in the cell wall after extraction with hot SDS solution. The strongly reduced YFP fluorescence in cell walls after treatment with hot SDS must therefore be mainly due to a loss of fluorescence by partial denaturation of YFP. The SDS-resistant incorporation into the cell wall identifies AC3362 as a biosilica-associated protein.

**Figure 6:**
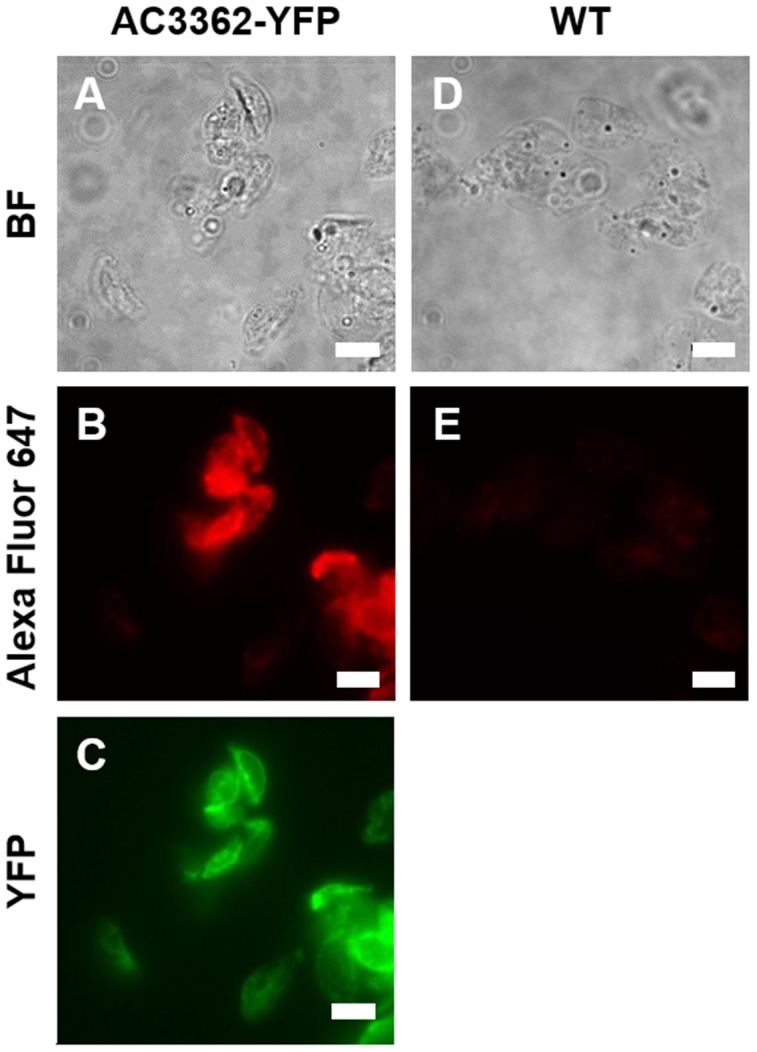
Ammonium fluoride extraction of cell walls. The ammonium fluoride insoluble materials from the cell walls of a AC3362-YFP expressing transfomant clone (**A-C**)) and from wild type (**D, E**) were analyzed by bright field microscopy (BF), by direct fluorescence microscopy (YFP), and by indirect immunofluorescence microscopy (Alexa Fluor 647) using an anti-YFP primary antibody and an Alexa Fluor 647-labeled secondary antibody. Bars = 5 µm.

**Figure 7:**
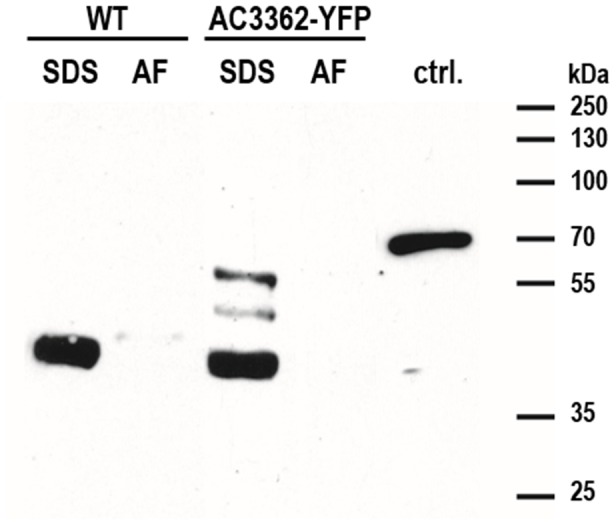
Western blot probed with an anti-YFP antibodies. The extracts obtained from identical amounts of either wild type (WT) or AC3362-YFP bearing cell walls by treatment with a hot SDS solution (SDS) or with an ammonium fluoride solution at pH 4–5 (AF) were loaded. In lane ‘ctrl.’ 100 ng recombinant Sil3-GFP (isolated from *E. coli*) was loaded.

Previously, two types of biosilica-associated proteins have been characterized: (i) proteins that become soluble when the silica is dissolved using an ammonium fluoride solution at pH 5 (e. g., silaffins; [35]), and (ii) proteins that are constituents of an organic matrix that remain insoluble after ammonium fluoride treatment (cingulins; [23]). To investigate the type of biosilica association of AC3362, isolated *A. coffeaeformis* cell walls bearing AC3362-YFP were resuspended in a solution of ammonium fluoride at pH 5. After dissolution of the silica, the insoluble material was recovered by centrifugation and investigated by fluorescence microscopy. The ammonium fluoride-insoluble material exhibited YFP fluorescence (Figure 6 A, C) thus indicating the presence of AC3362. This was confirmed by indirect immunolabeling using the same primary and secondary antibodies as above (Figure 6A, B). No fluorescence was observed when immunolabeling was performed with ammonium fluoride-insoluble material from wild type cell walls (Figure 6D, E), demonstrating the specificity of the immunolabeling procedure. To investigate whether any of the AC3362-YFP fusion protein was extracted during ammonium fluoride treatment, Western Blot analyses were performed using the anti-YFP antibody. No proteins were detected in the ammonium fluoride extracts from the AC3362-YFP bearing cell walls and wild type cell walls (Figure 7). A loading control (i. e., recombinant silaffin3-GFP purified from *E. coli*) indicated that the Western Blot procedure had worked (Figure 7) leaving the options that either the ammonium fluoride extract did not contain AC3362-YFP, or an insufficient amount of ammonium fluoride extract was loaded on the Western blot. The latter possibility could be ruled out by analyzing the hot SDS extract of AC3362-YFP bearing cell walls as described in the following paragraph.

In the hot SDS-extract from AC3362-YFP bearing cell walls three bands of ∼40 kDa, ∼45 kDa, and ∼55 kDa were recognized by the anti-YFP antibodies (Figure 7). The ∼40 kDa band was also present in the hot SDS-extract from wild type cell walls and thus resulted from non-specific cross-reaction with an unidentified protein. In contrast, the ∼45 kDa and ∼55 kDa bands were only present in the hot SDS-extract from the transformant cell walls, and thus must be related to the AC3362-YFP fusion protein. Considering the molecular mass of YFP (27 kDa) it can be concluded that the ∼45 kDa and ∼55 kDa proteins in the SDS extract contain ∼150 and ∼250 amino acids, respectively, of the C-terminal end of AC3362 provided that posttranslational modifications are absent. The immunofluorescence data shown above (see Figure 5) have demonstrated that hot SDS treatment extracts only a very small fraction of the AC3362-YFP fusion protein from the cell walls. This small amount could be detected in the Western Blot experiment indicating the high sensitivity of the method. As no YFP fusion protein was detected in the ammonium fluoride extract that was prepared from the same amount of cell walls, it was concluded that the vast majority of AC3362-YFP molecules is present in the ammonium fluoride-insoluble material.

Altogether the data from fluorescence microscopy and Western Blot analyses (see Figures 5–7) suggest that the majority of the AC3362-YFP fusion protein is a constituent of a biosilica-associated, insoluble organic matrix. The small amount of biosilica-associated YFP fusion protein extractable by hot SDS solutions is composed of relatively short fragments of AC3362 (≤23% and ≤38%) that may lack the domain(s) required for incorporation into the ammonium fluoride insoluble matrix. Determining the apparent molecular mass of the AC3362-YFP fusion protein in the ammonium fluoride insoluble organic matrix has not been possible, because attempts to solubilize this material have so far failed. Regarding itsincorporation in an insoluble organic matrix AC3362 resembles cingulins rather than silaffins.

## Discussion

Here we describe the identification of the first cell surface protein, AC3362, from *A. coffeaeformis*, a model species for studying diatom underwater adhesion to surfaces [14]–[16]. AC3362 was identified by screening a normalized *A. coffeaeformis* transcriptome database, which has been established in the present study (note: during the course of this work additional transcriptome databases for *A. coffeaeformis* have been published by other groups [40]). The database screen was performed using a novel amino acid composition-based bioinformatics screening software that we have developed here and made publicly available (http://vergil.chemistry.gatech.edu/cgi-bin/proteomics.py). The screening parameters were based on proteins that mediate underwater adhesion in marine mussels, which are highly enriched in both lysine and tyrosine residues [12], [13]. AC3362 contains two lysine- and tyrosine-rich domains (amino acids 95–146 and 147–325), but does not exhibit sequence similarity to mussel adhesion proteins.

Studies on the functional characterization of AC3362 relied on a genetic transformation system for *A. coffeaeformis* that has also been established in the present study. This enabled the expression of an AC3362-YFP fusion protein and investigation of its location by fluorescence microscopy using both direct imaging of the YFP fusion protein and indirect immunolabeling with anti-YFP antibodies. The data clearly indicate that AC3362 is not a component of the adhesive material that is secreted by the diatom cell. Instead, AC3362 is part of an insoluble organic matrix associated with the biosilica of the cell wall, similar to the cingulin-containing microrings recently described from *T. pseudonana* [23]. Several biosilica associated proteins, have been implicated in cellular biosilica formation, and the AC3362 protein resembles the cingulins from the diatom *T. pseudonana*, which are also rich in tyrosine residues [23], [35]–[37], [41].

As the AC3362-YFP fusion protein was accessible to antibody molecules in immunolabeling it appears to be partially or fully exposed on the biosilica surface, rather than completely embedded within the biosilica. Ammonium fluoride-insoluble organic matrices that are composed of proteins and polysaccharides and exposed on the biosilica surface have recently been identified in several diatoms (not including *Amphora* species) [42]. Tesson and Hildebrand argued that insoluble organic matrices embedded within diatom biosilica may not exist as they were unable to detect ammonium fluoride-insoluble organic matrices from acid hydrolyzed biosilica [42]. However, their data were inconclusive, because their argument was based on the resistance to acid hydrolysis of the biosilica-associated long-chain polyamines, whichare devoid of acid-labile bonds [38]. Therefore, it cannot be argued that silica-embedded organic matrices have to be resistant to acid hydrolysis conditions. In contrast, as biosilica is highly porous and hydrated it can be expected that protons can easily penetrate throughout the biosilica, and thus polysaccharide- and protein-based insoluble organic-matrices should become completely degraded under acid hydrolysis conditions, regardless of whether they are embedded within or exposed on the surface of biosilica. In future research, analysis by immunofluorescence microscopy, using the method described in the present study, may be able to validate whether the cingulin-containing organic matrix of *T. pseudonana* is embedded inside the biosilica or located on the biosilica surface.

It has been discussed that biosilica-associated organic matrices may have a role in biosilica formation, mechanical support of biosilica, the stabilization of biosilica against dissolution, or combinations of these functions [35], [42], [43]. One possibility to further investigate the function of the AC3362 containing organic matrix would be the generation of knock-down mutants. This technique is established for the diatoms *P. tricornutum* [44] and *T. pseudonana* [45], and should be also possible for *A. coffeaeformis* by utilizing the transformation system that has been established in this study.

The bioinformatics screen for mussel-like putative adhesion proteins in *A. coffeaeformis* has yielded three additional tyrosine-rich proteins (AC714, AC1077, AC3362). However, we were unable to detect by fluorescence microscopy the production of the resulting YFP fusion proteins in *A. coffeaeformis* transformants. Expression rate of the YFP fusion proteins may have been too low, the C-terminal YFP-tag may be proteolytically cleaved from the mature protein, or the YFP domain may have interfered with folding or stability of these tyrosine-rich proteins. Whatever the case, due to the presence of the encoding mRNAs it is reasonable to assume that the three proteins are produced in *A. coffeaeformis* wild type cells. Furthermore, the presence of an N-terminal signal peptide for co-translational ER import and the absence of transmembrane helices in each of these proteins strongly suggest that they become secreted into the medium, or are incorporated into the cell wall, or are targeted to another intracellular compartment. Protein AC714 contains repeats of the dipeptide KG (amino acids 419–599) which are also present in the cell wall protein AC3362 (amino acids 95–146, 326–357, 385–537). In the silaffin family of diatom proteins, lysine-rich repeats have been shown to promote association with the biosilica *in vivo*.

During the course of our work on the tyrosine-rich *A. coffeaeformis* proteins, a different study has provided evidence for the absence of Dopa in *A. coffeaeformis* [11]. As the adhesiveness of the tyrosine-rich proteins depends on conversion of tyrosine to Dopa residues [13], the tyrosine-rich *A. coffeaeformis* proteins AC714, AC1077, and 4076, which we have identified in the present study cannot be Dopa-dependent adhesion proteins. However, recently a Dopa-independent mechanism for underwater adhesion of the sandcastle worm has been proposed, which is based on complex coacervation [47]. Complex coacervation involves the aggregation of polyelectrolyte chains (here: proteins) resulting in liquid-liquid phase separation of a polyelectrolyte-rich and a polyelectrolyte-depleted aqueous phase [48], [49]. Aggregation of polyelectrolyte chains can be mediated through neutralization of oppositely charged polyelectrolytes or through the hydrophobic effect, yielding coacervate phases with low interfacial energies that are conducive to spreading on surfaces [47], [48]. AC714 and AC4076 exhibit domains with high densities of negative and positive charges (see Fig. 1) and might therefore self-aggregate or aggregate with other zwitterionic biomolecules. In contrast, AC1077 is mainly composed of uncharged amino acid residues with high proportions of tyrosine (17.6 mol-%) and proline (23.4 mol-%), which might promote hydrophobically driven complex coacervation. Investigating whether these unique proteins are involved in surface adhesion of *A. coffeaeformis* will require successful expression of tagged fusion proteins or the generation of specific antibodies to enable their localization, and establishing gene knockdown mutants followed by phenotype analysis.

The value of amino acid composition-based bioinformatics screens for the identification of novel diatom cell surface proteins has previously been demonstrated through the identification of cingulins [23] and a frustule-associated protein [32]. The amino acid-based screening method established in the present study substantially extends the possible search parameters and largely simplifies the procedure by providing a simple web-based interface. Therefore, we expect that amino acid-based screening methods will become increasingly used to identify proteins whose function is independent on a particular 3D fold and requires characteristic non-complex amino acid compositions.

## Supporting Information

Figure S1 **Alignments of the Y-rich proteins from** ***A. coffeaeformis*** **.** AC4076 (**A**), AC714 (**B**) and AC3362 (**C**) were aligned with their respective best BLAST hit using Clustal Omega (http://www.ebi.ac.uk/Tools/msa/clustalo/). Note that BLAST analyses did not yield hits for AC1077.
(DOCX)

Table S1 **Sequences of the Y-rich proteins.** Predicted N-terminal signal peptide sequences are underlined. Note that for AC203 5′-RACE PCR confirmed the sequence of amino acids 1-41, whereas the 3′-RACE PCR primers failed to yield a product.
(DOCX)

Table S2 **Oligonucleotide sequences of primers used in the present study.** Note that the 3′-RACE PCR primers for AC203 failed to yield a product.
(DOCX)

Table S3 **Amino acid composition of the Y-rich proteins from** ***A. coffeaeformis*** **.** Amino acid frequencis are shown in mol-%. Analyses were performed with ProtParam (http://web.expasy.org/protparam/). Note that the sequence of AC203 could be not verified by RACE PCR.
(DOCX)

Table S4 **PSI-BLAST hits for the Y-rich proteins from** ***A. coffeaeformis*** **.** Note that AC203 was not analyzed as its polypeptide sequence could not be verified by RACE PCR.
(DOCX)

Table S5 **Number of transformant clones analyzed by fluorescence microscopy.** The clones were selected on nourseothricin containing agar plates.
(DOCX)

File S1 **Domain analysis of the tyrosine-rich proteins.**
(DOCX)
