# Supplementary material for: A Tyrosine-Rich Cell Surface Protein in the Diatom Amphora coffeaeformis Identified through Transcriptome Analysis and Genetic Transformation
Source: PLoS One. 2014 Nov 5;9(11):e110369. doi: 10.1371/journal.pone.0110369 (PMC4220933; doi:10.1371/journal.pone.0110369)
Supplement: Figure S1 — Alignments of the Y-rich proteins from A. coffeaeformis . AC4076 (A), AC714 (B) and AC3362 (C) were aligned with their respective best BLAST hit using Clustal Omega (http://www.ebi.ac.uk/Tools/msa/clustalo/). Note that BLAST analyses did not yield hits for AC1077. (DOCX) [file pone.0110369.s001.docx]

**Figure S1. Alignments of the Y-rich proteins from *A. coffeaeformis*.** AC4076 (**A**), AC714 (**B**) and AC3362 (**C**) were aligned with their respective best BLAST hit using Clustal Omega (http://www.ebi.ac.uk/Tools/msa/clustalo/). Note that BLAST analyses did not yield hits for AC1077.

**A**

tr|G0W7Y6|G0W7Y6_NAUDC FFPEDYEDASADVVCYGSNLMITMGQASPGEM-LWVNALQSLEEGVNTIDQYIELTYSCS

AC4076 ------------MKLFGPFLPIALALSMTMNVSLAVKTTVGNNNKIDETDKDMTIQPVMS

: :* * *::. : :: * *:: . :: :: *: : : *

tr|G0W7Y6|G0W7Y6_NAUDC DTVASTIYTTEYSATTEYIIVDGYNSASALASSKTNACHHHYHYYWLDWFFY--------

AC4076 KTMG--------LRQGRRIMSSEWEPPRDLKKSSKKSS------KYDDWYYYHNYYYDKD

.*:. . *: . :: * .*..::. : **::*

tr|G0W7Y6|G0W7Y6_NAUDC ----------------YY---LLYRYYIHYWYRQYFHSRGHHPCRNTKHQRCHHYYHYYW

AC4076 HYYYPEHPQHDPYYYYYYYHHPEEPYYYYYYYHDYHHGKKSKGK-GKGKSSSDHYYYYYH

** ** :*:*::*.*.: : . :. ..***:**

tr|G0W7Y6|G0W7Y6_NAUDC L--DWFFYYYLLYRHYIHYWYRQYFHSRGHHPCRNTKHQRCHHYYHYYWLDWSFTTTYST

AC4076 PKDDYYYDYYHDKDHYYYYYYKDHYYGKGKKSMKKSKGKGSSHYDDYYYYYYY-HPEYKG

*::: ** ** :*:*:::::.:*:: :::* : . ** .**: : *.

**B**

gi|357624935|gb|EHJ75523.1| ----------------------MMCLHRGNRSVHKP------RTDLTRLFCYLRYISTVT

AC714 THKPTYKPTYKPTYKPTYKPTYKPTYKPTPKPAPKPAPKPTPKHCKERPICIPRQAGNDD

: : . ** : * :* * ..

gi|357624935|gb|EHJ75523.1| SLEGG-------GGFEHKSTQQASLVQ---------------------------------

AC714 DGNAGGTDDGNAGGTDDTGTDDKDKDDKDKDDKDKDDKEKEGNRRKLGSGVYPEVRRTYW

. :.* ** :...*:: . :

gi|357624935|gb|EHJ75523.1| -CASDSTTHTNVKIFS------------TNMLRLSTFCMLLALAIAQSGYEYNKPGRPFG

AC714 TAPAKYHYHDDYNYYHDDDYYYYYDDSVDDVYHGLPYCDELPTVSPVSG------GGPTS

. :. * : : : :: : :* * . ** * * .

gi|357624935|gb|EHJ75523.1| TTTPSSRPGYKPGQTAAYPGSSYPTTSQNGEYTPSSLGTTPNYPGFNRPQTGYPGQTPGG

AC714 TAAPTPTGGTS---STAAPSKEDKKDKDKMTRRVRSVGISSTYPDLRHLKVGPKPDDDDD

*::*: * . ::* *... . .:: *:* : .** :.: :.* :

gi|357624935|gb|EHJ75523.1| PTGPIAGPGGNYPDQGGKYPGKGGSYPGQGGNYPGQGSNYPGQGGNYPGQGGNLPGQGGN

AC714 --LPW-----CDEIKGKGYKGKGASYKGQGGNYKGQGGNYKGKGAIYKGQGGSYKGKGAS

* :* * ***.** ****** ***.** *:*. * ****. *:*..

gi|357624935|gb|EHJ75523.1| YPGQGSNYPGQGGNYPGQ----GGNLPGQGGNYPGQGSNYPGQGGNYPGQ----GGNLPG

Ac714 YKGKGDSYKGKGASYKGKDKSKGDSYKGKGGGYKGKGDSYKGKGGSYKGKDKSKGDSYKG

* *:*..* *:*..* *: * . *:** * *:*..* *:**.* *: * . *

gi|357624935|gb|EHJ75523.1| QGGNYPGQGSNYPGQGGNYPGQ--------------------GGNLPGQGGNYPGQASNY

AC714 KGGDYKGKGDSYKGKGGSYKGKDKGKGDSYKGGSYKGKDKSKGDSYKGKGGSYKGKGDNY

:**:* *:*..* *:**.* *: * . *:**.* *:..**

gi|357624935|gb|EHJ75523.1| PGQGSNYPAQGQTPERPGFGPGGPGFDNSGAYDNGDYSAIPGEPDKDYPILSTIPETSFR

AC714 KGKGDSYKGNGDSYK---------GYEKE--------------TSHY-------------

*:*..* .:*:: : *:::. .:

gi|357624935|gb|EHJ75523.1| CDAQPYPGYYADIETRCQVFHVCANNITYDFLCPNGTIFSQEYFVCVWWNQFDCNSAPSF

AC714 --------YYAKH-----------------------------------------------

***.

**C**

gi|328351261|emb|CCA37661.1| MVSLQTLFAAFLVSTCVAAPQTLTSPTGENSPVAIAEWER--TIPASGLVDSKIAENPTK

AC3362 -MKFSSAIL-ALLPFCALAQE-LFNNGGNDVP-NLSQFAAADSQALGGLENVKEQ-----

:.:.: : *: *. * : * . *:: * :::: : .** : *

gi|328351261|emb|CCA37661.1| ADTAAEKRTTVDFVAERQTGRPSANAAENPDSRPVQPNQPAQPLEPSQASQLDESVQ-TF

AC3362 ---------AQSLDRDRKTGLTRETINNEEDEEELV------EVEEE-ENEVDQESRALW

: .: :*:** . :: *.. : :* . .::*:. : :

gi|328351261|emb|CCA37661.1| QASQSLS---SQPT-------EPNQSLLANEPAQNANGGPTNNPVGKPP-GKPTDKPTDE

AC3362 WSWKGKGEGKGKGKGKGKGYSYKGSSDYKGKGYADYKGGSTSDYKGAPPTPHPTPKPTPK

: :. . .: . .* : : :** *.: * ** :** *** :

gi|328351261|emb|CCA37661.1| PTNKPTGKPTDKPTDKPTDKPTDKPTDKPTDKPT----GKPTDKPTDKPTDKPTDKPTDK

AC3362 PTHKPTYKPTHKPTYKPTYKPTYKPTYKPTYKPTYKPTYKPTHKPTYKPTYKPTYKPTYK

**.*** ***.*** *** *** *** *** *** ***.*** *** *** *** *

gi|328351261|emb|CCA37661.1| PTDKPTDKPTDKPTDKPTDKPTDKPTDKPTDKPTDKPTDKPTDKPTDKPTDKPTDKPTDK

AC3362 PTYKPTYKPTYKPTNKPTYKPTYKPTYKPTYKPTPKPSPHPTKKPTYKPTYKPTYKPTPK

** *** *** ***:*** *** *** *** *** **: :**.*** *** *** *** *

gi|328351261|emb|CCA37661.1| ----PTDKPTDKPTDKPTDKPTDKPTDKPTDKPTDKPTDKPTDKPTGKPTANGQTNG-QT

AC3362 PSPHPTKKPTYKPTYKPTPKPSPHPTPKPSPHPTKKPTPKPSPHPTPVPAK-GKGKGKGA

**.*** *** *** **: :** **: :**.*** **: :** *: *: :* :
