# Supplementary material for: A Tyrosine-Rich Cell Surface Protein in the Diatom Amphora coffeaeformis Identified through Transcriptome Analysis and Genetic Transformation
Source: PLoS One. 2014 Nov 5;9(11):e110369. doi: 10.1371/journal.pone.0110369 (PMC4220933; doi:10.1371/journal.pone.0110369)
Supplement: Table S1 — Sequences of the Y-rich proteins. Predicted N-terminal signal peptide sequences are underlined. Note that for AC203 5′-RACE PCR confirmed the sequence of amino acids 1-41, whereas the 3′-RACE PCR primers failed to yield a product. (DOCX) [file pone.0110369.s002.docx]

Table S1. Sequences of the Y-rich proteins. Predicted N-terminal signal peptide sequences are underlined. Note that for AC203 5’-RACE PCR confirmed the sequence of amino acids 1-41, whereas the 3’-RACE PCR primers failed to yield a product.

| AC4076 (218 amino acids) | |
| --- | --- |
| 1  61  121  181 | MKLFGPFLPI ALALSMTMNV SLAVKTTVGN NNKIDETDKD MTIQPVMSKT MGLRQGRRIM  SSEWEPPRDL KKSSKKSSKY DDWYYYHNYY YDKDHYYYPE HPQHDPYYYY YYYHHPEEPY  YYYYYYHDYH HGKKSKGKGK GKSSSDHYYY YYHPKDDYYY DYYHDKDHYY YYYYKDHYYG  KGKKSMKKSK GKGSSHYDDY YYYYYYHPEY KGKSRREA |
| AC1077 (448 amino acids) | |
| 1  61  121  181  241  301  361  421 | MKFAFLLSLL ATAEALNLGF KPKQEVKENK VKGEEEQRFL PGTDDYAYYP PQPVYYGCGG  KMYGGKMYAG INCPPVPGPA PVPAPTPDYP PPGPGDYPPP GPGDYYPPGP GSYYPGYGGG  GYYPYYPPAP APVPAGKMYG SGGSCGGKMY GGPCPAPAPY PYPAPAPYPY PGYGYGGGYG  YGGGYGYGGG YGYGGGYGYG GGYGGGYYGP GYYPGYYGGG YGYGGGYGYG GGYGYGGGYG  YGGGYGYGGG YYPYYPYYPP APAPVPVPAP TPAGKMYGGG SMSGGSMDGA SCGGKMYGGP  CPAPVPAPVP APTPPYYPPY VPPYYPPYVP PYYPPSYPPS GPICPPVAGG KMYGGKMYGS  TTPGPCYYPP APAPAPYPWP YPPAPYPWPY PPAPVPAPVP SPVPVPVPVP VPAPAPCGKM  YGGKMYGTAG CAGSGKMYGS RKLSEEKL |
| AC714 (613 amino acids) | |
| 1  61  121  181  241  301  361  421  481  541  601 | MIWKGSLVSF LFVSSSWVSV AVHVRSPNTD PSLTTVATTG AHHDEENVRS LYEQKKTYAS  GDHYKMVPHD DDYYNYYYYD DYKYYAKGKG KTMKSNGKGK GMSSSSKGKG KGYSTKGKGA  YSSGKGKGAS YKGYSKGASS YKGYYKGKGG YSAPKPTYKP TYKPTYKPTY KPTYKPTYKP  THKPTYKPTY KPTYKPTYKP TYKPTYKPTP KPAPKPAPKP TPKHCKERPI CIPRQAGNDD  DGNAGGTDDG NAGGTDDTGT DDKDKDDKDK DDKDKDDKEK EGNRRKLGSG VYPEVRRTYW  TAPAKYHYHD DYNYYHDDDY YYYYDDSVDD VYHGLPYCDE LPTVSPVSGG GPTSTAAPTP  TGGTSSTAAP SKEDKKDKDK MTRRVRSVGI SSTYPDLRHL KVGPKPDDDD DLPWCDEIKG  KGYKGKGASY KGQGGNYKGQ GGNYKGKGAI YKGQGGSYKG KGASYKGKGD SYKGKGASYK  GKDKSKGDSY KGKGGGYKGK GDSYKGKGGS YKGKDKSKGD SYKGKGGDYK GKGDSYKGKG  GSYKGKDKGK GDSYKGGSYK GKDKSKGDSY KGKGGSYKGK GDNYKGKGDS YKGNGDSYKG  YEKETSHYYY AKH |
| AC3362 (666 amino acids) | |
| 1  61  121  181  241  301  361  421  481  541  601  661 | MKFSSAILAL LPFCALAQEL FNNGGNDVPN LSQFAAADSQ ALGGLENVKE QAQSLDRDRK  TGLTRETINN EEDEEELVEV EEEENEVDQE SRALWWSWKG KGEGKGKGKG KGKGYSYKGS  SDYKGKGYAD YKGGSTSDYK GAPPTPHPTP KPTPKPTHKP TYKPTHKPTY KPTYKPTYKP  TYKPTYKPTY KPTYKPTHKP TYKPTYKPTY KPTYKPTYKP TYKPTYKPTN KPTYKPTYKP  TYKPTYKPTP KPSPHPTKKP TYKPTYKPTY KPTPKPSPHP TKKPTYKPTY KPTPKPSPHP  TPKPSPHPTK KPTPKPSPHP TPVPAKGKGK GKGADYKGKG GEGASYKGAK GYAPEPQCSA  HPDCAHLHGD CCPTTDGVYL FCCSLYEWTK GKGADYKGKG SDYKGKGKGK GKGKGADYKG  KGAPAPEPAK GKGAPAPEPA KGKGAPAPEP AKGKGAPAPE PAKGKGAEYK GKGAEYKGKG  AEYKGKGAPA PEPAKGKGAP APEPAKGKGA PAPAKGKGAP VPAKGKGAPV PAKGKGGSGS  CLHAEYYAVQ PDGTPVSDLS KASLDPGQTW SFSGAVYDKI GGEIVGHNYE LCTRINHGEM  WICEGNYVDL YGCSGQLTWE GPYSDATFTG LYTITGGTGD FVDAGGKIMG EFTYDGNYSY  RTMYVE |
| AC203 (336 amino acids) | |
| 1  61  121  181  241  301 | MILRPIWLLG ALPICSNIVV ASVDSKGESE SQALRGRSEI EQEQDSASTK KHNWLIGSIN  DLRKRSQQSD GSADGKVNQI ALDNLQLTED ADAYYYYYYY EPDYYYNHEY YYDHYFSEGK  GGASKGKGKG GHSYYSHEPG YYYTNDNYFS KGKGGTSKGK GKGAYSYKGK GVVVGKGKGK  GKGKGKGKGT TKGFWKRTTA TPQSTPPSSS IPTTTPPTQS PSARVVQRTF SPVIRATPRP  SPRPTPRPTP RRTPRPTVRS TSQPTPRSTP QPSPRPTPRS YTSSHSTSHF ASYASTYAST  YASTYASTYA STYASTYAST YASTYASTYA STYAST |
