# Supplementary material for: A Tyrosine-Rich Cell Surface Protein in the Diatom Amphora coffeaeformis Identified through Transcriptome Analysis and Genetic Transformation
Source: PLoS One. 2014 Nov 5;9(11):e110369. doi: 10.1371/journal.pone.0110369 (PMC4220933; doi:10.1371/journal.pone.0110369)
Supplement: Table S2 — Oligonucleotide sequences of primers used in the present study. Note that the 3′-RACE PCR primers for AC203 failed to yield a product. (DOCX) [file pone.0110369.s003.docx]

**Table S2.** **Oligonucleotide sequences of primers used in the present study.** Note that the 3’-RACE PCR primers for AC203 failed to yield a product.

| **Oligonucleotide sequences for RACE PCR** | | |
| --- | --- | --- |
| **Gene ID** | **3’-RACE PCR (5´-3´)** | **5’-RACE PCR (5´-3´)** |
| **AC203** | AC203_3f: CACTCCACGTCCCACTTTGCGTC | AC203_5r: GTTCGATTTCGCTTCGCCCGCG |
|  | AC203_4f: CGGCTAGAGTCGTTCAGAGAAC | AC203_6r: TTTCCGTCTGCACTGCCGTCGG |
|  | AC203_7f: TTTTGGAAACGTACCACTGCCAC |  |
|  | AC203_8f: CGCGGGCGAAGCGAAATCGAAC |  |
|  | AC203_9f: CCGACGGCAGTGCAGACGGAAA |  |
| **AC1077** | AC1077_3f: ATCTCGTAAACTGTCAGAAG | AC1077_5r: AGTACCAGGCAAGAAACGTTG |
|  | AC1077_4f: ACTGCAGGATGCGCCGGATCC | AC1077_6r: GGTTCAAAGCTTCGGCAGTTGC |
| **AC714** | AC714_3f**:** ACGAAAAGGAGACATCCCATTAC | AC714_5r: CATGGACTGCCACTGACACCC |
|  | AC714_4f: CCATTACTACTACGCCAAACAC | AC714_6r: GTGAGGCACCATTTTGTAATG |
| **AC3362** | AC3362_7f: GGTCGACCAAGAAAGCCGTGC | AC3362_5r: ACCTGTCTTGTGGTCTCGATC |
|  | AC3362_8f: CGTGAAGGAACAGGCCCAAAG | AC3362_6r: CCAACTCCACCAGAGAGCACG |
| **AC4076** | AC4076_3f: CCACCCGGAATACAAAGGCAAG | AC4076_5r: GGTCATATCCTTGTCTGTTTCG |
|  | AC4076_4f: GGCAAGAGCCGTCGTGAGGCT | AC4076_6r: CGGGGTGGTTCCCCTTCGGAC |
|  | | |
| **Oligonucleotide sequences for amplification of the full-length genes** | | |
| **Gene ID** | **Forward oligonucleotide (5´-3´)** | **Reverse oligonucleotide (5´-3´)** |
| **AC714** | GATTGATATCATGATTTGGAAAGGCTCCTTGG | AATCTCTAGAGTGTTTGGCGTAGTAGTAATGG |
| **AC1077** | GATTTACGTAATGAAGTTCGCGTTTTTGCTTTCGCTC | AATCTCTAGAAAGCTTTTCTTCTGACAGTTTAC |
| **AC3362** | GATTGATATCATGAAGTTTTCGAGTGCAATTCTTGC | AATCTCTAGACTCAACGTACATGGTTCGGTAC |
| **AC4076** | TCCGTACGTAATGAAGCTGTTTGGCCCCTTTC | AATCTCTAGAAGCCTCACGACGGCTCTTGCC |
