# Supplementary material for: A Tyrosine-Rich Cell Surface Protein in the Diatom Amphora coffeaeformis Identified through Transcriptome Analysis and Genetic Transformation
Source: PLoS One. 2014 Nov 5;9(11):e110369. doi: 10.1371/journal.pone.0110369 (PMC4220933; doi:10.1371/journal.pone.0110369)
Supplement: Table S3 — Amino acid composition of the Y-rich proteins from A. coffeaeformis . Amino acid frequencis are shown in mol-%. Analyses were performed with ProtParam (http://web.expasy.org/protparam/). Note that the sequence of AC203 could be not verified by RACE PCR. (DOCX) [file pone.0110369.s004.docx]

**Table S3.** **Amino acid composition of the Y-rich proteins from *A. coffeaeformis*.** Amino acid frequencis are shown in mol-%. Analyses were performed with ProtParam (http://web.expasy.org/protparam/). Note that the sequence of AC203 could be not verified by RACE PCR.

|  | **AC4076** | **AC1077** | **AC714** | **AC3362** | **AC203** |
| --- | --- | --- | --- | --- | --- |
| **Ala** | 1.8 | 7.6 | 4.1 | 8.3 | 7.4 |
| **Arg** | 2.8 | 0.4 | 2.0 | 0.9 | 5.7 |
| **Asn** | 2.3 | 0.7 | 2.1 | 2.0 | 2.4 |
| **Asp** | 8.7 | 1.3 | 10.0 | 3.8 | 3.3 |
| **Cys** | 0.0 | 2.2 | 0.7 | 1.7 | 0.3 |
| **Gln** | 1.4 | 0.7 | 0.8 | 1.5 | 3.6 |
| **Glu** | 3.7 | 1.8 | 2.0 | 5.6 | 3.0 |
| **Gly** | 6.0 | 23.4 | 16.3 | 13.2 | 9.2 |
| **His** | 7.3 | 0.0 | 2.3 | 2.3 | 2.1 |
| **Ile** | 1.8 | 0.4 | 1.0 | 1.2 | 3.0 |
| **Leu** | 3.2 | 2.0 | 1.6 | 3.3 | 3.0 |
| **Lys** | 13.3 | 4.2 | 18.4 | 15.5 | 7.4 |
| **Met** | 3.7 | 3.1 | 0.8 | 0.6 | 0.3 |
| **Phe** | 0.9 | 0.9 | 0.3 | 1.4 | 1.5 |
| **Pro** | 5.5 | 23.4 | 6.5 | 14.3 | 8.3 |
| **Ser** | 8.3 | 3.3 | 9.0 | 4.7 | 14.3 |
| **Thr** | 2.8 | 1.8 | 6.4 | 8.7 | 11.0 |
| **Trp** | 0.9 | 0.4 | 0.7 | 1.1 | 0.9 |
| **Tyr** | 23.9 | 17.6 | 12.2 | 8.0 | 10.1 |
| **Val** | 1.8 | 4.5 | 2.9 | 2.4 | 3.3 |
