# Supplementary material for: A Tyrosine-Rich Cell Surface Protein in the Diatom Amphora coffeaeformis Identified through Transcriptome Analysis and Genetic Transformation
Source: PLoS One. 2014 Nov 5;9(11):e110369. doi: 10.1371/journal.pone.0110369 (PMC4220933; doi:10.1371/journal.pone.0110369)
Supplement: Table S4 — PSI-BLAST hits for the Y-rich proteins from A. coffeaeformis . Note that AC203 was not analyzed as its polypeptide sequence could not be verified by RACE PCR. (DOCX) [file pone.0110369.s005.docx]

**Table S4**. **PSI-BLAST hits for the Y-rich proteins from *A. coffeaeformis*.** Note that AC203 was not analyzed as its polypeptide sequence could not be verified by RACE PCR.

| **Gene ID** | **Sequence identity (%)** | **e-value** | **Matched length (aa)** | **Best PSI-BLAST Hit** |
| --- | --- | --- | --- | --- |
| **AC4076** | 29 | 3e-34 | 103 | G0W7Y6_NAUDC *Naumovozyma dairenensis* (strain ATCC 10597) (Unknown function) |
| **AC714** | 41 | 3e-36 | 179 | EHJ75523.1 putative Collagen alpha-1V chain *Danaus plexippus*. |
| **AC3362** | 67 | 1e-68 | 185 | Uncharacterized protein C2orf16 [*Komagataella pastoris* CBS 7435] |
| **AC1077** | - | - | - | No hit |
