# Supplementary material for: A Tyrosine-Rich Cell Surface Protein in the Diatom Amphora coffeaeformis Identified through Transcriptome Analysis and Genetic Transformation
Source: PLoS One. 2014 Nov 5;9(11):e110369. doi: 10.1371/journal.pone.0110369 (PMC4220933; doi:10.1371/journal.pone.0110369)
Supplement: Table S5 — Number of transformant clones analyzed by fluorescence microscopy. The clones were selected on nourseothricin containing agar plates. (DOCX) [file pone.0110369.s006.docx]

Table S5. Number of transformant clones analyzed by fluorescence microscopy. The clones were selected on nourseothricin containing agar plates.

| **Gene ID** | **Number of clones analyzed** |
| --- | --- |
| AC1077 | 27 |
| AC714 | 57 |
| AC3362 | 155 |
| AC4076 | 113 |
