# Supplementary material for: A Tyrosine-Rich Cell Surface Protein in the Diatom Amphora coffeaeformis Identified through Transcriptome Analysis and Genetic Transformation
Source: PLoS One. 2014 Nov 5;9(11):e110369. doi: 10.1371/journal.pone.0110369 (PMC4220933; doi:10.1371/journal.pone.0110369)
Supplement: File S1 — Domain analysis of the tyrosine-rich proteins. (DOCX) [file pone.0110369.s007.docx]

**File S1.** **Domain analysis of the tyrosine-rich proteins.**

**AC4076.**

| **Domain (aa)** | **Amino acid sequence** | **Y (%)** | **D+E (%)** | **K+R (%)** | **D+E+K+R (%)** | **Comment** |
| --- | --- | --- | --- | --- | --- | --- |
| 1-23 | MKLFGPFLPIALALSMTMNVSLA | 0 | 0 | 4 | 4 | Signal peptide |
| 24-70 | V**K**TTVGNNN**K**IDETD**K**DMTIQPVMS**K**TMGL**R**QG**RR**IMSSEWEPP**R**DL | 0 | 15 | 17 | 32 |  |
| 71-79 | **KK**SS**KK**SS**K** | 0 | 0 | 56 | 56 | S-rich (44 % S) |
| 80-131 | YDDWYYYHNYYYD**K**DHYYYPEHPQHDPYYYYYYYHHPEEPYYYYYYYHDYHH | 48 | 17 | 2 | 19 | H-rich (17 % H) |
| 132-145 | G**KK**S**K**G**K**G**K**G**K**SSS | 0 | 0 | 43 | 43 | KG-repeat |
| 146-179 | DHYYYYYHP**K**DDYYYDYYHD**K**DHYYYYYY**K**DHYY | 53 | 21 | 9 | 29 | H-rich (15 % H) |
| 180-195 | G**K**G**KK**SM**KK**S**K**G**K**GSS | 0 | 0 | 44 | 44 | KG-repeat |
| 196-218 | HYDDYYYYYYYHPEY**K**G**K**S**RR**EA | 39 | 18 | 18 | 34 |  |

**AC1077**

| **Domain (aa)** | **Amino acid sequence** | **Y (%)** | **D+E (%)** | **K+R (%)** | **D+E+K+R (%)** | **Comment** |
| --- | --- | --- | --- | --- | --- | --- |
| 1-15 | MKFAFLLSLLATAEAL | 0 | 7 | 7 | 13 | Signal peptide |
| 16-40 | LNLGF**K**P**K**QEV**K**EN**K**V**K**GEEEQ**R**FL | 0 | 20 | 24 | 44 |  |
| 41-171 | PGTDDYAYYPPQPVYYGCGG**K**MYGG**K**MYAGINCPPVPGPAPVPAPTPDYPPPGPGDYPPPGPGDYYPPGPGSYYPGYGGGGYYPYYPPAPAPVPAG**K**MYGSGGSCGG**K**MYGGPCPAPAPYPYPAPAPYPYP | 17 | 4 | 3 | 7 | P-rich (30 %) |
| 172-260 | GYGYGGGYGYGGGYGYGGGYGYGGGYGYGGGYGGGYYGPGYYPGYYGGGYGYGGGYGYGGGYGYGGGYGYGGGYGYGGGYYPYYPYYPP | 37 | 0 | 0 | 0 | G[GY(G)_3_Y] repeats |
| 261-314 | APAPVPVPAPTPAG**K**MYGGGSMSGGSMDGASCGG**K**MYGGPCPAPVPAPVPAPTP | 4 | 2 | 4 | 6 | P-rich (26 %) |
| 315-416 | PYYPPYVPPYYPPYVPPYYPPSYPPSGPICPPVAGG**K**MYGG**K**MYGSTTPGPCYYPPAPAPAPYPWPYPPAPYPWPYPPAPVPAPVPSPVPVPVPVPVPAPAP | 17 | 0 | 2 | 2 | P-rich(42 %) |
| 417-448 | CG**K**MYGG**K**MYGTAGCAGSG**K**MYGS**RK**LSEE**K**L | 9 | 6 | 19 | 25 |  |

**AC714**

| **Domain (aa)** | **Amino acid sequence** | **Y (%)** | **D+E (%)** | **K+R (%)** | **D+E+K+R (%)** | **Comment** |
| --- | --- | --- | --- | --- | --- | --- |
| 1-21 | MIWKGSLVSFLFVSSSWVSVA | 0 | 0 | 5 | 5 | Signal peptide |
| 22-85 | VHV**R**SPNTDPSLTTVATTGAHHDEENV**R**SLYEQ**KK**TYASGDHY**K**MVPHDDDYYNYYYYDDY**K**YY | 17 | 17 | 9 | 26 |  |
| 86-151 | A**K**G**K**G**K**TM**K**SNG**K**G**K**GMSSSS**K**G**K**G**K**GYST**K**G**K**GAYSSG**K**G**K**GASY**K**GYS**K**GASSY**K**GYY**K**G**K**GGY | 12 | 0 | 27 | 27 | Repetitive, S-rich (18 %) |
| 152-223 | SAP**K**PTY**K**PTY**K**PTY**K**PTY**K**PTY**K**PTY**K**PTH**K**PTY**K**PTY**K**PTY**K**PTY**K**PTY**K**PTY**K**PTP**K**PAP**K**PAP**K**PTP**K** | 17 | 0 | 25 | 25 | KPTY-repeats |
| 224-260 | HC**K**E**R**PICIP**R**QAGNDDDGNAGGTDDGNAGGTDDTGT | 0 | 22 | 8 | 30 |  |
| 261-288 | DD**K**D**K**DD**K**D**K**DD**K**D**K**DD**K**E**K**EGN**RRK**LG | 0 | 46 | 39 | 86 | DK-repeats |
| 289-340 | SGVYPEV**RR**TYWTAPA**K**YHYHDDYNYYHDDDYYYYYDDSVDDVYHGLPYCDE | 27 | 23 | 6 | 29 |  |
| 341-371 | LPTVSPVSGGGPTSTAAPTPTGGTSSTAAPS | 0 | 0 | 0 | 0 | PST-rich |
| 372-418 | **K**ED**KK**D**K**D**K**MT**RR**V**R**SVGISSTYPDL**R**HL**K**VGP**K**PDDDDDLPWCDEI | 0 | 26 | 24 | 50 | DK-rich |
| 419-613 | **K**G**K**GY**K**G**K**GASY**K**GQGGNY**K**GQGGNY**K**G**K**GAIY**K**GQGGSY**K**G**K**GASY**K**G**K**GDSY**K**G**K**GASY**K**G**K**D**K**S**K**GDSY**K**G**K**GGGY**K**G**K**GDSY**K**G**K**GGSY**K**G**K**D**K**S**K**GDSY**K**G**K**GGDY**K**G**K**GDSY**K**G**K**GGSY**K**G**K**D**K**G**K**GDSY**K**GGSY**K**G**K**D**K**S**K**GDSY**K**G**K**GGSY**K**G**K**GDNY**K**G**K**GDSY**K**GNGDSY**K**GYE**K**ETSHYYYA**K**H | 14 | 9 | 28 | 36 | DSYK-repeats |

**AC3362**

| **Domain (aa)** | **Amino acid sequence** | **Y (%)** | **D+E (%)** | **K+R (%)** | **D+E+K+R (%)** | **Comment** |
| --- | --- | --- | --- | --- | --- | --- |
| 1-17 | MKFSSAILALLPFCALA | 0 | 0 | 6 | 6 | Signal peptide |
| 18-94 | QELFNNGGNDVPNLSQFAAADSQALGGLENV**K**EQAQSLD**R**D**RK**TGLT**R**ETINNEEDEEELVEVEEEENEVDQES**R**AL | 0 | 28 | 8 | 36 |  |
| 95-146 | WWSW**K**G**K**GEG**K**G**K**G**K**G**K**G**K**GYSY**K**GSSDY**K**G**K**GYADY**K**GGSTSDY**K**GAPPTP | 11 | 8 | 23 | 31 | KG-repeats |
| 147-325 | HPTP**K**PTP**K**PTH**K**PTY**K**PTH**K**PTY**K**PTY**K**PTY**K**PTY**K**PTY**K**PTY**K**PTY**K**PTH**K**PTY**K**PTY**K**PTY**K**PTY**K**PTY**K**PTY**K**PTY**K**PTN**K**PTY**K**PTY**K**PTY**K**PTY**K**PTP**K**PSPHPT**KK**PTY**K**PTY**K**PTY**K**PTP**K**PSPHPT**KK**PTY**K**PTY**K**PTP**K**PSPHPTP**K**PSPHPT**KK**PTP**K**PSPHPTPVPA | 13 | 0 | 23 | 23 | KPTY-repeats |
| 326-355 | **K**G**K**G**K**G**K**GADY**K**G**K**GGEGASY**K**GA**K**GYAPE | 9 | 10 | 27 | 37 | KG-repeats |
| 356-386 | PQCSAHPDCAHLHGDCCPTTDGVYLFCCSLY | 0 | 10 | 0 | 10 | C-rich |
| 387-537 | EWT**K**G**K**GADY**K**G**K**GSDY**K**G**K**G**K**G**K**G**K**G**K**GADY**K**G**K**GAPAPEPA**K**G**K**GAPAPEPA**K**G**K**GAPAPEPA**K**G**K**GAPAPEPA**K**G**K**GAEY**K**G**K**GAEY**K**G**K**GAEY**K**G**K**GAPAPEPA**K**G**K**GAPAPEPA**K**G**K**GAPAPA**K**G**K**GAPVPA**K**G**K**GAPVPA**K**G**K**GG | 4 | 8 | 24 | 32 | DYKGKG-repeats |
| 538-666 | SGSCLHAEYYAVQPDGTPVSDLS**K**ASLDPGQTWSFSGAVYD**K**IGGEIVGHNYELCT**R**INHGEMWICEGNYVDLYGCSGQLTWEGPYSDATFTGLYTITGGTGDFVDAGG**K**IMGEFTYDGNYSY**R**TMYVE | 9 | 13 | 4 | 17 | Y-rich |
